# Effect of Massage on the TLR4 Signalling Pathway in Rats with Neuropathic Pain

**DOI:** 10.1155/2020/8309745

**Published:** 2020-12-16

**Authors:** Qian Wang, Jing Lin, Peng Yang, Yingye Liang, Dongming Lu, Kailong Wang, Wei Gan, Jianping Fu, Zhenbao Gan, Mingchen Ma, Pingting Wu, Fengshi He, Jun Pang, Hongliang Tang

**Affiliations:** ^1^Department of Acupuncture, The First Affiliated Hospital of Guangxi University of Chinese Medicine, Nanning, Guangxi 530000, China; ^2^Guangxi University of Chinese Medicine, Nanning, Guangxi 530200, China; ^3^Department of Massage, The First Affiliated Hospital of Guangxi University of Chinese Medicine, Nanning, Guangxi 530000, China

## Abstract

This study set out to investigate the effect of massage on the Toll-like receptor 4 (TLR4) signalling pathway in the dorsal root ganglia of rats that had undergone spinal nerve ligation (SNL), with the hypothesis that massage could be used as an analgesic. Forty female SD rats were randomly divided into 5 groups: the control group, sham-operated group, model group, sham massage group, and massage group. There were 8 rats in each group. SNL rat models were established in the model group, sham massage group, and massage group. Rats in the sham-operated group underwent surgery to expose the vertebral nerves, but no further procedures were performed. The control group consisted of intact animals. The rats in the massage group underwent massage using a massage simulation machine once a day for 14 d in succession; the hind limbs of the rats in the sham massage group were gently touched with a cloth bag once a day for 14 continuous days. The rats in the control group, the sham-operated group, and the model group did not receive any intervention and were observed for 14 d. Paw withdrawal thermal latency (PWTL) and paw withdrawal mechanical threshold (PWMT) of rats in each group were detected 1 d before modelling and at 1, 3, 7, and 14 d after modelling. Fourteen days after modelling, the expression levels of TLR4, IRAK1, TRAF6, TNF-*α*, and IL-6 were detected in all rats. The PWTL and PWMT of SNL rats were decreased, while these parameters were elevated after massage. SNL rats showed higher levels of TLR4, IRAK1, TRAF6, IL-6, and TNF-*α*, and massage effectively lowered the expression levels of these molecules. Inhibiting activation of the TLR4 signalling pathway, which can reduce the release of inflammatory factors, may be one mechanism by which massage treats neuropathic pain.

## 1. Introduction

The NeuPSIG of the International Society for Pain Research (IASP) defines neuropathic pain (NPP) as pain caused by a lesion or disease of the somatosensory system [1]. In clinical practice, the incidence of NPP is high, and its duration is long; NPP seriously affects patients' ability to work, quality of life, and mental state and, thus, imposes a high cost globally [2]. It is estimated that 6.9% to 10% of the world's population experiences chronic pain (such as inflammatory pain, cancer pain, and neuropathological pain) [3]. In the United States alone, $650 billion is spent annually on treating chronic pain [4].

The ultimate goal of NPP treatment is to reduce pain in patients and improve quality of life by minimizing side effects and reducing medical costs. The treatment of NPP usually includes drug therapy, neuromodulation therapy, and Chinese medicine. Medications include nonsteroidal anti-inflammatory drugs, opioid analgesics, anticonvulsants, and antidepressants [5, 6]. However, the side effects of drugs are plentiful and difficult to avoid. Although neuromodulation therapy is recommended as a first-line treatment for NPP due to its low risk, studies have shown that the efficacy of pulse therapy, which is widely used to treat clinical NPP, gradually decreases with use [7, 8]. Chinese massage is a common analgesic method of treating chronic pain; its analgesic effect and safety have been verified in clinical practice, and it is often used for pain treatment. However, due to a lack of research into its efficacy and mechanism, no serious recommendations have been made for its use in pain relief [9]. Therefore, it is timely to explore the mechanisms underlying the analgesic effects of massage.

Toll-like receptor 4 (TLR4) is a type of transmembrane signal transduction receptor that mediates inherent immunity and identifies pathogen-related pattern molecules (PAMPs). Several studies have confirmed that TLR4 plays an important role in NPP. Interleukin 1 receptor kinase (IRAK1) is widely expressed in a variety of cells and is a receptor protein located in the cytoplasm and nuclei. Its main function is to participate as a receptor protein kinase in TLR and other signalling pathways, causing the expression of immune response-related genes, which have important regulatory effects on inflammatory responses. As a downstream factor in the TLR4 signalling pathway, IRAK1 binds with the tumour necrosis factor (TNF) to form a receptor complex, which activates the MAPK family through a series of reactions. P38MAPK is a member of the MAPK family, and the P38MAPK signalling pathway is activated and can eventually regulate the release of inflammatory media to participate in NPP [10, 11]. In our preliminary study [12], we confirmed that the expression of molecules related to the P38MAPK signalling pathway is elevated in rats with NPP, but that the expression of molecules related to the P38MAPK signalling pathway is inhibited after massage, causing a reduction in the release of inflammatory factors and an analgesic effect. Based on this, we speculated that the mechanism by which massage decreases the expression of molecules related to the P38MAPK signalling pathway is through inhibiting molecular expression downstream of the P38MAPK signalling pathway via inhibition of the TLR4 signalling pathway.

To verify the efficacy of massage in reducing chronic pain, we developed a spinal nerve ligation (SNL) model to simulate NPP in rats and examined the expression of molecules related to the TLR4 signalling pathway after massage.

## 2. Materials and Methods

### 2.1. Animals

Forty SPF female SD rats weighing 250–300 g and aged 8–10 weeks were provided by Hunan Slake Jingda Experimental Animal Co., Ltd. (Hunan, China) (production license number: SCXK (Xiang) 2016-0002, license number: SYXK (Gui) 2014-0003). The housing conditions were appropriate, ventilation was adequate, and suitable temperature and light conditions were provided. The animals were offered water and an ad-lib standard chow diet, and the bedding was replaced daily.

### 2.2. Reagents and Materials

QPCR Reverse Kit Prime Script^TM^ RT Master Mix (DRR036A) and real-time PCR kit TB Green^TM^ Premix ExTaqTMII (Tli RNaseH Plus) (RR820A) were purchased from TaKaRa Bao Biotechnology (Dalian) Co., Ltd., Dalian, China. The IL-6 ELISA Kit and TNF-alpha ELISA Kit were purchased from Jiangsu Enzyme Standard Biotech Co., Ltd., Jiangsu, China.

### 2.3. SNL Model

Experiments began after a 7-day period of adaptation. The animals were randomly divided into the control group (*n* = 8), sham-operated group (*n* = 8), model group (*n* = 8), sham massage group (*n* = 8), and massage group (*n* = 8). The selected model of chronic pain was an NPP model involving left L5 SNL, which was performed according to the surgical procedure described by Kim and Chung et al. [13]. After conventional anaesthesia, the left side of the posterior superior spine of each rat was located, and the skin was shaved to approximately 3 cm either size of the razor, and routine disinfection was performed. A vertical incision was made between the left side of the posterior superior spine and the spine, which was about 2 cm long and parallel to the spine; two parallel spinal nerves were identified after the inner fascia and muscle were separated, and the nerves were ligated with 5-0 absorbable catgut suture. Saline was used to clean the wound before suturing, and the wound was closed layer by layer.

### 2.4. Model Criteria

The rats exhibited spontaneous lifting of the left hind paws, limb suspension, and lameness and prevented the left hind paws from touching the ground when walking. Successful SNL model establishment was based on a reduction in paw PWTL and PWMT and a change in the walking gait.

The rats in the model group, sham massage group, and massage group underwent the SNL modelling. No surgery was performed on the control group; in the sham-operated group, the nerves were exposed, but the ligation procedure was not performed.

### 2.5. Massage Therapy

After binding the rat with a cloth bag, a massage simulation machine (Chinese Republic Invention Patent No. ZL 200710187403.1) was used to stimulate the following acupoints: Huantiao (GB 30), Fengshi (GB 31), and Yanglingquan (GB 34) from the gallbladder Channel of Foot Shaoyang (both sides); the simulation manipulations used were the point method, stroke method, and kneading method, and the force was 4 N. A massage head with a diameter of 10 mm was selected. Each acupoint was massaged for 3 min and each method was for 1 min; each rat underwent 18 min of massage every day for 14 continuous days.

The rats in the sham massage group were restrained with cloth bags, and the hind limbs of the rats were gently touched for 18 min every day for 14 continuous days. The control group, the sham-operated group, and the model group were fed and cared for as previously described; they were observed for 14 d.

### 2.6. PWTL

A hot pad apparatus was preheated to a temperature of 55°C, and the air temperature was set to 25°C. A rat was placed in the test cage (to prevent the rat from escaping), and a timer was started simultaneously. When the rat tried to lift or lick its foot, the timer was stopped, and the time was recorded as the PWTL [7]. Three consecutive measurements were made with an interval of 5 min between each treatment; the average time was calculated as the PWTL, which represented the reaction time of the rat to heat stimulation. The experimental environment was kept quiet to avoid frightening the rats. To avoid burning the rats, a cutoff time of 20 sec was set. PWTL was tested 1 d before modelling and 1, 3, 7, and 14 d after modelling.

### 2.7. PWMT

Each group of rats was placed in a cage in a quiet environment 10 min before the experiment. Then, the left foot of the rat was stimulated using von Frey filaments from the bottom of the cage; 4, 6, 8, 10, 15, 26, 60, and 100 g von Frey filaments were used. The experiment was stopped when a positive result was recorded. Each von Frey filament test was repeated 5 times. If a rat lifted or licked its paw 3 or more times in response to a filament, the reaction was considered positive; otherwise, it was considered negative. The force of the filament that elicited a positive reaction was considered the PWMT [14]. Each test was completed when a positive reaction was observed. The next trial started after a rest period of 5 min, and three trials were performed; the mean value was considered the final result. If a positive reaction was not elicited by a von Frey filament with a force of less than 100 g, the PWMT of the rat was considered 100 g. The PWMT of each group of rats was detected 1 d before modelling and 1, 3, 7, and 14 d after modelling.

### 2.8. QPCR Analysis

The mRNA levels of TLR4, IRAK1, and TRAF6 were detected by QPCR of the L3-5 dorsal root ganglia of rats. The rats were sacrificed by cervical dislocation following anaesthesia with sodium pentobarbital (50 mg/kg). Specimens from the lumbar area of the rats were collected, and the skin and soft tissue were removed after exposing the spinal mastoid process. The spinal nerve was pulled out away from the spinal column, and then, the L3-5 dorsal root ganglia of the rats were removed, immediately placed in liquid nitrogen, and transferred to −80°C for cryopreservation. For total RNA extraction, 1000 *μ*L TRIzol reagent was added to the tissue, and the tissue was ground for homogenisation and then reverse transcribed into cDNA. According to the instructions of the TaKaRa real-time PCR kit and TB Green^TM^ Premix Ex TaqTM II (Tli RNaseH Plus) (RR820A), target gene mRNA transcription was measured with beta-actin as the internal control. Primers for TLR4, IRAK1, and TRAF6 were designed according to the sequences in GenBank. The primers were synthesized by TaKaRa Bao Bioengineering; the primer sequence is shown in Table 1. The reaction conditions were as follows: pre-denaturation at 95°C for 30 sec, 40 cycles of 95°C for 5 sec, and 60°C for 30 sec. Melting curve analysis was performed after the end of the full cycle. The PCR products were evaluated, and relative expression was calculated by formula (2^−△△ct^).

### 2.9. ELISA Analysis

The expression levels of IL-6 and TNF-*α* from the L3-5 dorsal root ganglia were detected according to the instructions of an IL-6 ELISA Kit and TNF-alpha ELISA Kit.

### 2.10. Statistical Analysis

The data were analysed using Statistical Package for Social Sciences (SPSS) software v22.0. Changes in the PWMT and PWTL were analysed by repeated-measures ANOVA with a within-subjects factor of time (5 levels: before surgery and 1, 3, 7, and 14 d after surgery) and a between-subjects factor of group (5 levels: the treatment groups) with post hoc LSD or Dunnett's T3 test. The expression of TLR4, IRAK1, TRAF6, TNF-*α*, and IL-6 was analysed using one-way analysis of variance (ANOVA) with post hoc LSD or Dunnett's T3 test. The results are expressed as the mean ± standard deviation, and *P* < 0.05 was considered significant.

## 3. Results

### 3.1. Changes in the PWMT and PWTL

Repeated measurements were taken, and significant differences in PWMTs (*F* (16,112) = 5.369, *P* < 0.001) and PWTLs (*F* (16,112) = 17.279, *P* < 0.001) between the groups and time points were found. No differences were found among the groups before model development (*P* > 0.05). After model development, the PWMT and PWTL of the control group were higher than those of all the other groups (*P* < 0.05) subjected to model development. On day 1 and day 3 after surgery, the PWMT and PWTL of the massage group were not different from those of the model group and sham massage group, but significant differences started to emerge on day 7 and day 14 after surgery, suggesting that massage started to be efficacious 7 d after the procedure.

**Figure 1:**
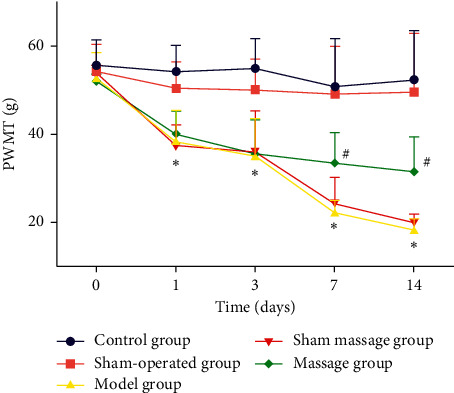
PWMTs of the groups 1 day, 3 days, 7 days, and 14 days after surgery. ^*∗*^*P* < 0.05 compared with the control group; ^#^*P* < 0.05 compared with the model group.

**Figure 2:**
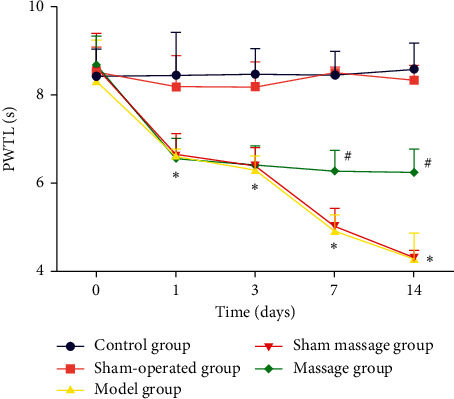
PWTLs of the groups 1 day, 3 days, 7 days, and 14 days after surgery. ^*∗*^*P* < 0.05 compared with the control group; ^#^*P* < 0.05 compared with the model group.

In the control and sham-operated groups, no significant differences were found between the different time points; in the model group, the PWMT and PWTL were lower at 1, 3, 7, and 14 d after model development than before model development, suggesting that model development was successful; in the sham massage group, and the PWMT and PWTL showed a decreasing trend over time (*P* < 0.05). In the massage group, the same trend was found, but no significant difference was detected (*P* > 0.05), suggesting that massage can effectively inhibit decreases in the PWMT and PWTL, as shown in Tables 2 and 3, and Figures 1 and 2.

### 3.2. QPCR Results of TLR4, IRAK1, and TRAF6 Expression among Groups

On the 14th day after model development, the mRNA expression of TLR4, IRAK1, and TRAF6 in the control group was not significantly different when compared with that in the sham-operated group (*P* > 0.05); the mRNA expression levels of TLR4, IRAK1, and TRAF6 in the control group and sham-operated group were lower than those in the model group, sham massage group, and massage group (*P* < 0.05).

**Figure 3:**
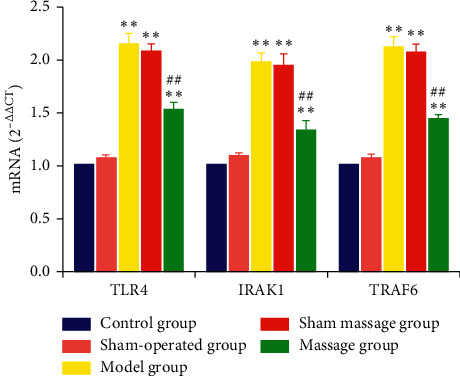
TLR4, IRAK1, and TRAF6 mRNA expression 14 days after surgery. ^*∗∗*^*P* < 0.01 compared with the control group; ^##^*P* < 0.01 compared with the model group.

The mRNA expression levels of TLR4, IRAK1, and TRAF6 were similar between the model group and the sham massage group (*P* > 0.05), whereas mRNA expression levels of TLR4, IRAK1, and TRAF6 in the massage group were lower than those in the model group and the sham massage group (*P* < 0.05), as shown in Table 4 and Figure 3.

### 3.3. ELISA Results of TNF-*α* and IL-6 among Groups

**Figure 4:**
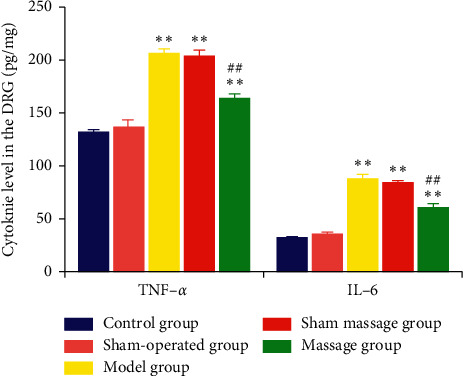
TNF-*α* and IL-6 expression 14 days after surgery. ^*∗∗*^*P* < 0.0; compared with the control group; ^##^*P* < 0.01 compared with the model group.

On the 14th day after model development, the expression levels of TNF-*α* and IL-6 in the control group were not significantly different from those in the sham-operated group (*P* > 0.05), whereas the expression levels of TNF-*α* and IL-6 in the control group and the sham-operated group were lower than those in the model group, the sham massage group, and the massage group (*P* < 0.01). Moreover, the expression levels of TNF-*α* and IL-6 in the model group were similar to those in the sham massage group (*P* > 0.05). Finally, the expression levels of TNF-*α* and IL-6 in the model group and the sham massage group were higher than those in the massage group, and the difference was statistically significant (*P* < 0.01), as shown in Table 5 and Figure 4.

## 4. Discussion

The salient results of this study were as follows: (1) PWTL and PWMT of SNL rats were decreased, but PWTL and PWMT were increased after massage; and (2) the expression of TLR4, IRAK1, TRAF6, IL-6, and TNF-*α* were increased in SNL rats, and the expression levels decreased after massage.

TLR4 is a transmembrane signal transduction receptor that mediates innate immunity and recognizes pathogen-associated molecular patterns (PAMPs). PAMPs such as bacteria, viruses, and fungi stimulate immune cells to express these receptor molecules. TLR4 recognizes and binds with bacterial lipopolysaccharides (LPS)/endotoxins on the cell membrane, which activates nuclear transcription factors and increases the expression of inflammatory mediators through two signalling pathways [15]. The MyD88- (myeloid differentiation primary response 88-) dependent pathway is one of these pathways; TLR4 and MyD88 can promote the IRAK family after binding. Phosphorylated IRAKs dissociate from MyD88, interact with TRAF6, a member of the TRAF family, and further activate TAK1 as MAPKKK. TAK1 can activate the MAPK family and promote the nuclear translocation of NF-*κ*B through a series of reactions. P38MAPK is a member of the MAPK family. The MAPK signalling pathway is related to nerve injury [10, 11]. NF-*κ*B can regulate the synthesis and secretion of early inflammatory mediators, such as TNF-*α* and IL-6. In recent years, several studies have reported that NPP is a result of the joint action of inflammatory cytokines and chemokines mediated by immune cells in the nervous system [16]. Inflammatory mediators and various cytokines can be detected at injury sites, in the surrounding area, and even in the plasma.

The current results showed that the expression levels of TLR4, IRAK1, TRAF6, TNF-*α*, and IL-6 were increased significantly on the 14th day after SNL model initiation; this suggests that the TLR4 signalling pathway was activated in NPP, which further induced the occurrence of inflammatory reactions, leading to the perception of pain. After 14 d of massage intervention, the expression of TLR4, IRAK1, TRAF6, TNF-*α*, and IL-6 decreased significantly, suggesting that massage deregulated the TLR4 signalling pathway, which, in turn, inhibited the inflammatory response and, thus, alleviated the perception of pain in rats. According to the theory of gate control [17], the activation of peripheral nerve fibres can excite glial cells (SGs) in the posterior horn of the spinal cord; SGs inhibit the activity of first transfer cells (T cells), which can shut the “gate” that transmits pain and inhibit the transmission of pain. Interestingly, the stimulatory signal generated by massage can be transmitted to the posterior horn of the spinal cord as a noninjurious sensation along coarse fibres. In other words, the mechanism by which massage could have alleviated pain in SNL rats may have been related to the ability of the stimulation produced by the massage technique to activate SGs at the level of the spinal cord posterior horn, inhibit T cells, close the gate, and inhibit pain signal transmission. The results showed that massage can inhibit the transmission of the TLR4 signalling pathway and reduce the release of inflammatory factors, which has also been demonstrated in the previous research [12].

## 5. Conclusions

In conclusion, massage may inhibit the TLR4 signalling pathway and reduce inflammatory factors. This could be one mechanism of massage-induced pain relief and provides a reference for the mechanism research of massage-induced pain relief.

## Figures and Tables

**Table 1:** Primer sequences.

Gene	Primer sequence (5′ to 3′)
TRAF6-F	GCCCATGCCGTATGAAGAGA
TRAF6-R	ACTGAATGTGCAGGGGACTG
IRAK1-F	GCTCCCAGACCCATTCTGAG
IRAK1-R	CTCTGGGCTGGCTTGATGG
TLR4-F	CTCACAACTTCAGTGGCTGGATTTA
TLR4-R	GTCTCCACAGCCACCAGATTCTC

**Table 2:** PWMTs of the rats. The data are shown as $\bar{X}\pm s$ (*g*, *n* = 8). ^*∗*^*P* < 0.05 compared with the control group; ^#^*P* < 0.05 compared with the model group.

Group	Before surgery	1 day after surgery	3 days after surgery	7 days after surgery	14 days after surgery
Control	55.75 ± 5.87	54.33 ± 6.06	55.29 ± 6.60	51.04 ± 10.84	52.46 ± 11.22
Sham-operated	54.33 ± 6.06	50.58 ± 6.02	50.08 ± 7.26	49.17 ± 10.91	49.63 ± 13.34
Model	52.92 ± 5.86	38.33 ± 7.14^*∗*^	35.00 ± 8.74^*∗*^	22.33 ± 2.77^*∗*^	18.33 ± 2.47^*∗*^
Sham massage	53.88 ± 6.66	37.38 ± 4.91^*∗*^	35.96 ± 9.43^*∗*^	24.25 ± 6.03^*∗*^	20.04 ± 1.90^*∗*^
Massage	52.00 ± 6.80	40.17 ± 5.25^*∗*^	35.54 ± 7.99^*∗*^	33.58 ± 6.94^*∗*#^	31.71 ± 7.76^*∗*#^

**Table 3:** PWTLs of the rats. The data are shown as $\bar{X}\pm s$ (g, *n* = 8). ^*∗*^*P* < 0.05 compared with the control group; ^#^*P* < 0.05 compared with the model group.

Group	Before surgery	1 day after surgery	3 days after surgery	7 days after surgery	14 days after surgery
Control	8.42 ± 0.61	8.45 ± 0.97	8.47 ± 0.59	8.45 ± 0.53	8.56 ± 0.61
Sham-operated	8.52 ± 0.55	8.19 ± 0.70	8.17 ± 0.57	8.50 ± 0.50	8.34 ± 0.32
Model	8.30 ± 0.96	6.63 ± 0.15^*∗*^	6.30 ± 0.33^*∗*^	4.94 ± 0.34^*∗*^	4.30 ± 0.58^*∗*^
Sham massage	8.58 ± 0.82	6.67 ± 0.47^*∗*^	6.39 ± 0.41^*∗*^	5.05 ± 0.39^*∗*^	4.32 ± 0.18^*∗*^
Massage	8.69 ± 0.62	6.57 ± 0.45^*∗*^	6.41 ± 0.41^*∗*^	6.28 ± 0.46^*∗*#^	6.24 ± 0.53^*∗*#^

**Table 4:** TLR4, IRAK1, and TRAF6 mRNA expression. The data are shown as $\bar{X}\pm s$; *n* = 8. ^*∗∗*^*P* < 0.0 compared with the control group;^##^*P* < 0.01 compared with the model group.

Group	TLR4	IRAK1	TRAF6
Control	1.00 ± 0.00	1.00 ± 0.00	1.00 ± 0.00
Sham-operated	1.06 ± 0.04	1.08 ± 0.03	1.06 ± 0.05
Model	2.14 ± 0.11^*∗∗*^	1.97 ± 0.10^*∗∗*^	2.11 ± 0.11^*∗∗*^
Sham massage	2.07 ± 0.08^*∗∗*^	1.93 ± 0.13^*∗∗*^	2.06 ± 0.09^*∗∗*^
Massage	1.52 ± 0.08^*∗∗*^^##^	1.32 ± 0.11^*∗∗*^^##^	1.43 ± 0.05^*∗∗*^^##^

**Table 5:** The expression of TNF-*α* and IL-6 in each group on the 14th day after surgery. ^*∗∗*^*P* < 0.0 compared with the control group; ^##^*P* < 0.01 compared with the model group.

Group	TNF-*α*	IL-6
Control	130.75 ± 2.02	30.91 ± 0.57
Sham-operated	135.37 ± 8.28	33.46 ± 2.30
Model	206.04 ± 4.27^*∗∗*^	86.73 ± 5.06^*∗∗*^
Sham massage	202.67 ± 6.56^*∗∗*^	83.51 ± 1.20^*∗∗*^
Massage	162.85 ± 4.89^*∗∗*^^##^	59.06 ± 4.88^*∗∗*^^##^

## Data Availability

The data used to support the findings of this study are available from the corresponding authors upon request.
